# Discovery, Isolation, and Bactericidal Activity of a Cyclotide from *Spigelia anthelmia* L. (Loganiaceae)

**DOI:** 10.1021/acs.jnatprod.5c01216

**Published:** 2026-01-12

**Authors:** Toluwanimi E. Akinleye, Latifat O. Sidiq, Alfred Attah, Roland Hellinger, Lisa Pabi, Nermina Malanovic, Omonike O. Ogbole, Christian W. Gruber

**Affiliations:** † Center for Physiology and Pharmacology, Medical University of Vienna, Schwarzspanierstr. 17, Vienna 1090, Austria; ‡ Department of Pharmacognosy and Herbal Medicine, Faculty of Pharmaceutical Sciences, University of Ibadan, Ibadan 200284, Nigeria; § Institute of Molecular Biosciences, 64839 University of Graz, Humboldtstraße 50/III, Graz 8010, Austria; ∥ Department of Plant and Environmental Biology, Kwara State University, Malete 241103, Nigeria; ⊥ Department of Pharmacognosy and Drug Development, Faculty of Pharmaceutical Sciences, 108285 University of Ilorin, Ilorin 240003, Nigeria; # Field of Excellence BioHealth, University of Graz, Graz 8010, Austria; ∇ Bio TechMed Graz, Graz 8010, Austria

## Abstract

Cyclotides are plant-derived macrocyclic peptides stabilized by a cystine-knot motif, found in a limited number of angiosperm plants. This study reports the discovery of the cyclotide, Spat1, from *Spigelia anthelmia* (Loganiaceae), expanding the phylogenetic range of known cyclotide-producing plants. Spat1, a 30-residue bracelet-type cyclotide, was isolated, purified, and sequenced *de novo*. It demonstrated strong bactericidal activity against the Gram-positive *Bacillus subtilis* (LC_99.9_ = 20 μM) via rapid membrane disruption but showed no activity against *Staphylococcus aureus* or Gram-negative *Escherichia coli* (LC_99.9_ > 400 μM). The selective lack of activity against *S. aureus* is unusual for antimicrobial peptides. The data suggest that Spat1’s activity is independent of lipoteichoic acid (LTA) in *B. subtilis*, suggesting that its mechanism involves interactions with cytoplasmic membrane phospholipids. The lack of phosphatidylethanolamine (PE) in *S. aureus* membranes and Spat1’s weak binding to LTA, combined with its low net positive charge (+1), likely explains its inefficacy against this bacterial species. Structural modeling using AlphaFold AfCycDesign indicated that Spat1 adopts a cyclotide-typical β-sheet architecture and a 3_10_-helix within its loop regions. Overall, Spat1 broadens understanding of cyclotide diversity and evolution, highlighting their functional specialization and the convergent evolutionary pressures that shape their distribution across plant lineages.

## Graphical abstract

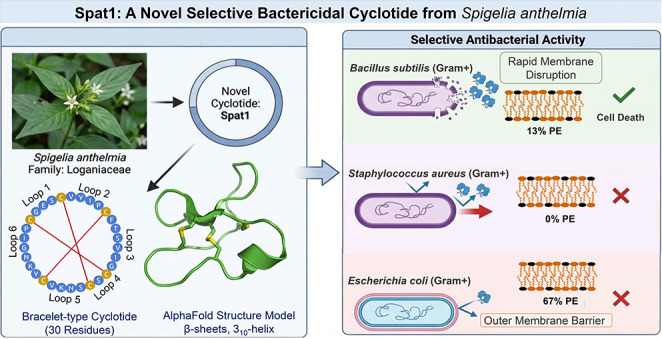

Cyclotides are well-defined, stable plant-derived circular mini-proteins (28–37 amino acids) featuring a conserved cyclic cystine-knot (CCK) motif formed by N–C terminal cyclization and disulfide bond formation.^1,2^ Activity loss upon linearization underscores the critical role of its structural fold.^3,4^ This compact architecture provides exceptional stability against thermal, chemical, and enzymatic degradation^5^ while enabling diverse bioactivities, making them promising scaffolds for drug development. Two structural classes exist: Möbius cyclotides possess a cis-proline in loop 5, causing backbone torsion, while bracelet cyclotides lack this twist in their fold.

As of July 2025, over 800 plant cyclotides have been cataloged in CyBase (https://www.cybase.org.au/), the dedicated cyclic peptide database.^6^ Despite this diversity, most are taxonomically restricted to single plant species, with few documented across phylogenetically distant families.

Cyclotides were first identified in *Oldenlandia affinis* (Rubiaceae) and subsequently documented across a few angiosperm families, revealing phylogenetically constrained distribution. Rubiaceae remains the richest source (>200 cyclotides), with confirmed occurrences in the tribes Psychotrieae and Hedyotieae. Many occurrences have been reported in Violaceae (primarily genus *Viola*),^7,8^ whereas they are sparsely distributed in Fabaceae (*Clitoria ternatea*),^9^ Solanaceae (*Petunia* spp.),^10^ Cucurbitaceae (less characterized cyclotide-like trypsin inhibitor peptides from *Momordica cochinchinensis*),^11^ and Poaceae (acyclotides in *Panicum laxum*),^12^ suggesting evolutionary convergence or horizontal gene transfer.^13^ So far, Rubiaceae (Gentianales) and Violaceae (Malpighiales) contain most known cyclotides.^14,15^

Concisely, cyclotides have so far been reported in just the above-mentioned six of the ∼416 angiosperm families (<2%). While less than half of angiosperm families have so far been phytochemically screened for cyclotides, their detection in only six appears to illustrate restricted distribution, which raises questions about their evolutionary origins. Phylogenetic evidence supports convergent evolution across lineages, likely driven by shared ecological pressures.^9,13,15^ Their primary role in plants has been described as defense molecules, evidenced by potent insecticidal, antimicrobial, and cytotoxic activities.

Cyclotides are exceptionally versatile bioactive peptides with diverse therapeutic applications, including antimicrobial, immunosuppressive, anticancer, and hemolytic activities.^16–19^ Their broad-spectrum antimicrobial action primarily involves microbial membrane disruption, causing cell lysis.^20–22^ For example, cycloviolacin O2 (cyO2) from *Viola odorata* exhibits potent activity against both Gram-positive (*S. aureus*, *B. subtilis*) and Gram-negative (*E. coli*) pathogens.^17^ This selective targeting of microbial over mammalian membranes stems from its amphipathic structure, enabling specific lipid bilayer interactions.^23–25^

Antimicrobial resistance (AMR) represents a critical global health threat, causing an estimated 4.95 million deaths in 2019, 1.27 million directly attributable to resistant bacteria.^26,27^ Without intervention, annual fatalities could reach 10 million by 2050, imposing $100 trillion in economic losses. Low- and middle-income nations face heightened vulnerability due to inadequate healthcare access, poor sanitation, and unregulated antibiotic use.^28^ This silent pandemic demands the urgent discovery of sustainably produced antimicrobials with unconventional mechanisms of action.

In this study, 10 plants from two orders and three families, viz. Gentianales (Gentianaceae and Loganiaceae) and Malpighiales (Euphorbiaceae) were extracted and screened for the presence of cyclotides using a mass spectrometry-based peptidomics approach. We report cyclotides in a previously unexplored family, i.e., Loganiaceae, and describe the antimicrobial effects of a novel cyclotide from *S. anthelmia*.

## Results and Discussion

### Small-Scale Peptidomics Screening Identification of Plant Peptides

Cysteine-rich peptides are gene-encoded ribosomally synthesized and post-translational modified peptides (RiPP) whose distribution is limited to a few angiosperm families, including the orders of Gentianales and Malpighiales. Since phylogenetically adjacent taxa often express similar genes, we explored 13 samples (10 species) from these two orders, i.e., leaves of *Euphorbia hirta*, *E. hyssopifolia*, *E. humifusa*, *Strychnos floribunda*, *S. inocua*, *S. spinosa*, *Rauvolfia vomitoria*, whole plant of *S. anthelmia*, leaf and root of *Anthocleista djalonensis* and *A. liebrechtsiana*, for the presence of cyclotides. All samples were solvent extracted, partially purified, enriched by solid-phase extraction (SPE) and analyzed by MALDI-TOF mass spectrometry. Mass peaks (2900–3900 Da) were observed in the peptide extract from *S. anthelmia* whole plant (SAW) (Figure 1A), but none of the other samples yielded any signals (*m*/*z* 2000–4000) corresponding to cyclotides (Figure S1 and Table S1, Supporting Information). Biochemical analysis of SAW resulted in +348 Da shift after treatment with iodoacetamide (carbamidomethylation of 6 Cys residues) and additional +18 Da after EndoGluC digestion (cyclotide backbone hydrolysis) (Figure 1A). The sample also exhibited late RP-HPLC eluting peaks (30–70 min retention time, RT) (Figure 1B), characteristic of hydrophobic cyclotides. This study targeted unexplored families from the orders Gentianales (Loganiaceae and Gentianaceae) and Malpighiales (Euphorbiaceae). Cyclotides were detected in *S. anthelmia* (Loganiaceae), but they were absent in *Strychnos* spp. (Loganiaceae) and *Anthocleista* spp. (Gentianaceae) (Figure S1, Supporting Information), which might support a hypothesis of convergent evolution of cyclotides across plant lineages.^13^

**Figure 1:**
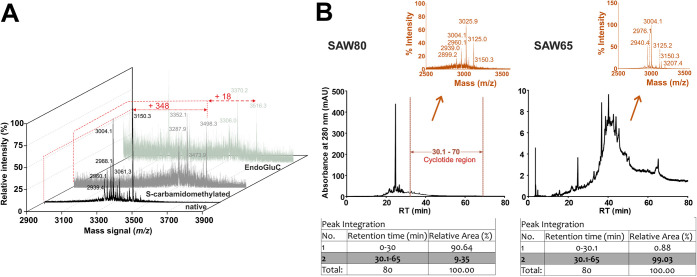
Detection of cyclotides in *Spigelia anthelmia*. (A) MALDI MS spectra showing the major mass signals as monoisotopic masses [M + H]^+^, with a +348 Da shift observed after carbamidomethylation and an additional +18 Da shift (+366 in total) following endopeptidase EndoGluC digestion, which implies a hydrolytic cleavage of glutamic acid that is usually conserved in cyclotides, leading to the circular opening of the cyclotides. (B) Analytical HPLC chromatogram showing peptide enrichment to improve solid-phase peptide extraction toward large-scale extraction and isolation after 10% buffer B wash and 80% buffer B elution (SAW80), and 30% buffer B wash and 65% elution (SAW65).

### Peptide Extraction and Characterization

Due to the weak peptide-containing peaks observed in analytical RP-HPLC (<10% peak area) (Figure 1B), the SPE protocol was optimized for peptide enrichment. Nonpeptide components eluted between 0 and 30 min RT, while peptides eluted between 30 and 70 min RT. Consequently, the SPE method was modified: a 30% buffer B wash replaced the original 10% wash, and elution used 65% buffer B (SAW65) instead of 80% (SAW80). This enrichment increased the peptide peak area substantially from 9.3% area-under-the-curve (SAW80) to 99.03% (SAW65) (Figure 1B).

### Bioassay-Guided Purification of Cyclotides

Cyclotides are defense molecules constituting the innate immune response in plants. These peptides act against diverse pathogenic microbes. To identify peptides with antimicrobial activity, we screened the isolated extract for rapid bactericidal action, as this method is not majorly influenced by components present in growth media (e.g., salts).^29–31^ Therefore, we performed an assay by incubating the peptide-enriched extract SAW65 with bacteria for 1 h in sodium phosphate buffer (PBS) and then determined bacterial survival by colony counting (Figure 2). Bactericidal assay revealed that SAW65 lacked activity against *E. coli* (LC_99.9_ ≥ 1000 μg/mL) but was active against *B. subtilis* (LC_99.9_ = 50 μg/mL) (Figure 2A). SAW65 separation yielded nine HPLC fractions, five of which (D–H) contained peptides (Figure 2B). Bioassay-guided fractionation on *B. subtilis* identified activity solely in SAW65 fraction G (LC_99.9_ = 50 μg/mL) (Figure 2B); other peptide fractions (D–F, H) were inactive against both *E. coli* and *B. subtilis* (Table S2, Supporting Information).

**Figure 2:**
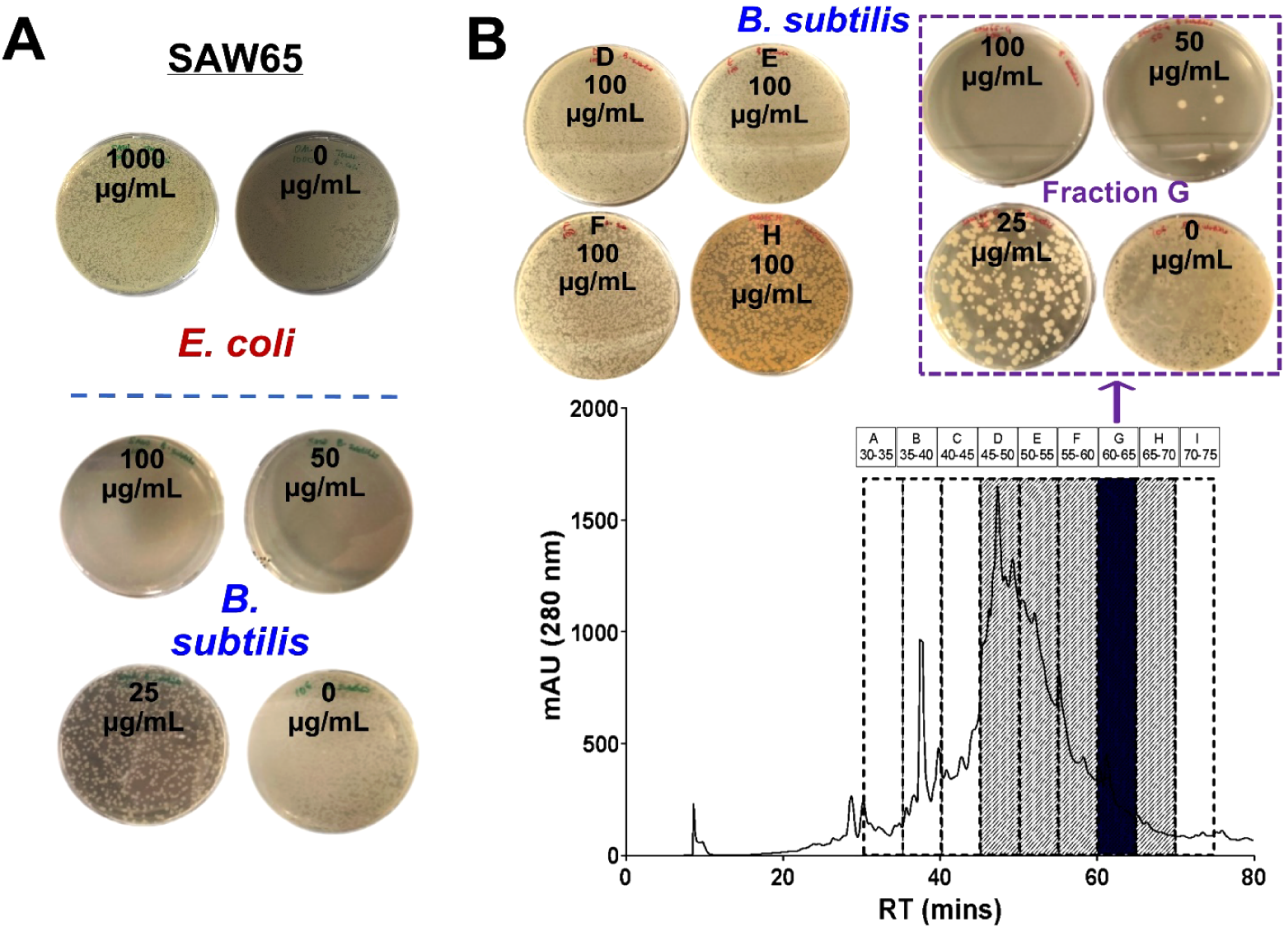
Fractionation and bactericidal effect of SAW65 peptide extract. (A) Bactericidal effects of fraction SAW65 on Gram-negative *E. coli* and *B. subtilis*, after 1 h incubation, and (B) preparative HPLC chromatogram exemplifying fractionation and bactericidal effects of peptide-containing HPLC fractions (D–H, concentration marked on plates) on Gram-positive bacteria of *B. subtilis*. Fraction G, highlighted in black in (B), maintained the bactericidal effect (dashed square) observed in the SAW65 peptide extract. The data presented are representative of at least three independent experiments, each yielding similar results.

Semipreparative HPLC purification of fraction G yielded purified Spat1 (Figure 3A); [M + H]^+^ = 3150.1 Da (Figure 3B). To investigate whether LTA – a highly anionic component of the Gram-positive bacterial cell envelope – functions as an initial interaction site for Spat1 and potentially influences its translocation across the membrane, we evaluated the peptide’s activity against a *B. subtilis* mutant strain lacking LTA. If LTA were a critical determinant for initial binding, its absence would be expected to reduce antimicrobial potency. However, Spat1 exhibited a comparable LC_99.9_ value against the LTA-deficient mutant, ΔLTA *B. subtilis* (LC_99.9_ = 20 μM) (Figure 3C), and the wild-type strain (LC_99.9_ = 20 μM) (Figure 3D), with no effect on *E. coli* (Figure 3E) or *S. aureus* (Figure 3F), indicating that LTA may not be required for the mode of action responsible for membrane permeability and bactericidal activity.

**Figure 3:**
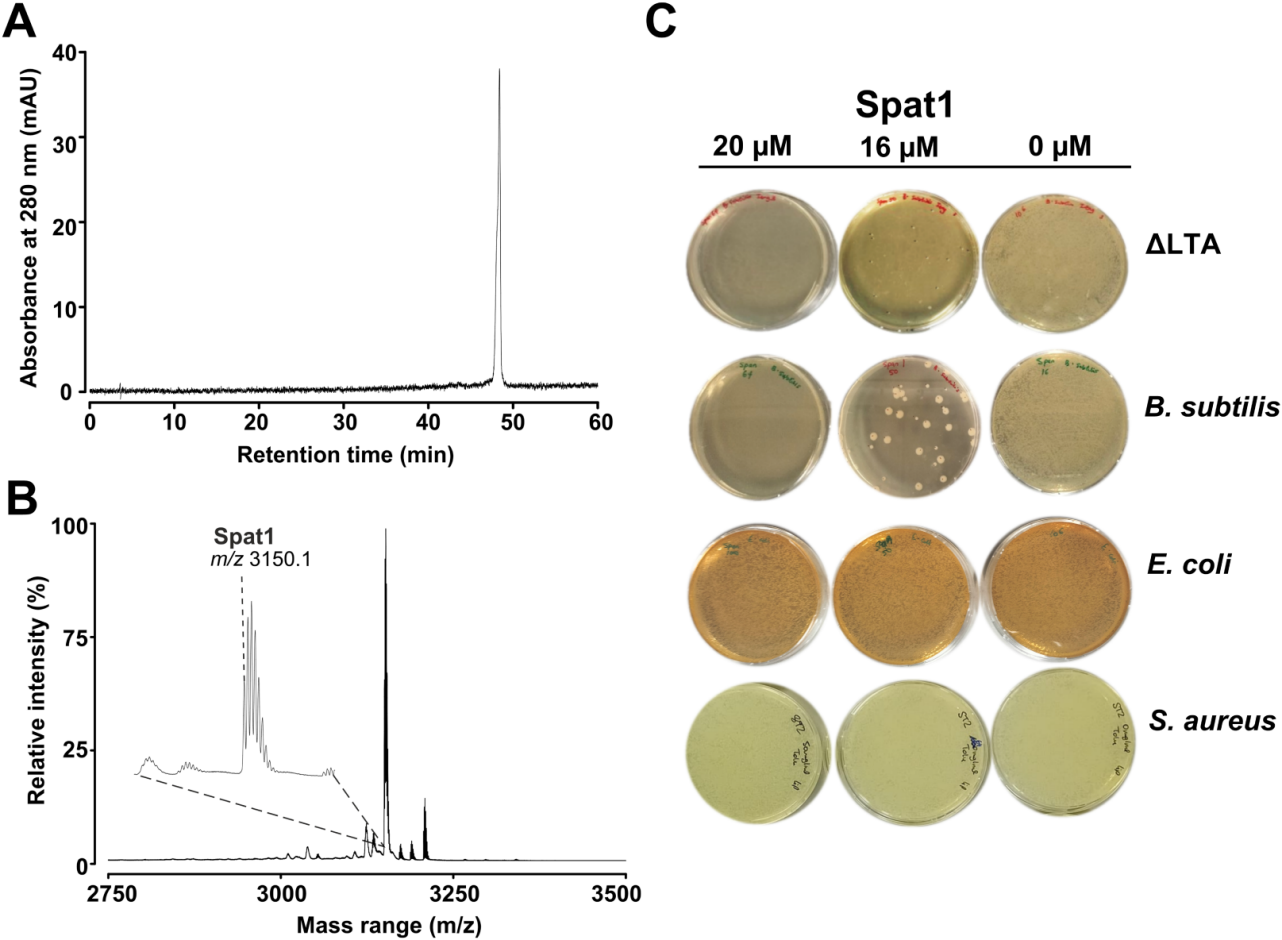
Purification and bactericidal effect of Spat1. (A) Analytical HPLC chromatogram of Spat1, (B) MALDI MS spectrum showing the mass signal of Spat1 as a monoisotopic mass [M + H]^+^, and (C) the bactericidal effect of Spat1 on ΔLTA *B. subtilis*, *B. subtilis*, *E. coli*, and *S. aureus*. After 1 h incubation with bacterial cells, Spat1 (≥95% purity) demonstrated LC_99.9_ at 20 μM against *B. subtilis* and LTA-deficient *B. subtilis* but no effect against Gram-negative *E. coli* and Gram-positive *S. aureus*. The data presented are representative of at least three independent experiments, each yielding similar results.

### Bacterial Membrane Permeability Effects of Cyclotides

Cyclotides demonstrate broad-spectrum antimicrobial activity, primarily by disrupting microbial membranes to induce cellular rupture and cell death. Within 60 s of exposure to LC_99.9_ concentrations, SAW65 (Figure 4A) and Spat1 (Figure 4B) caused swift membrane permeability in *B. subtilis*, as observed in a 2 h propidium iodide (PI) incubation assay of peptide and bacteria. Notably, the membrane-permeabilizing shift directly coincides with the bactericidal concentration range of both SAW65 and Spat1, indicating that this activity is lethal to the bacteria. Interestingly, the uptake of normally membrane-impermeable PI was induced to a similar extent as observed for the well-known membrane-active controls melittin and octenidine.^32,33^ Further, Spat1 (Figure S2, Supporting Information) showed low binding affinity with LTA, as evidenced by the low BODIPY-cadaverine displacement (Additional Methods, Supporting Information).

**Figure 4:**
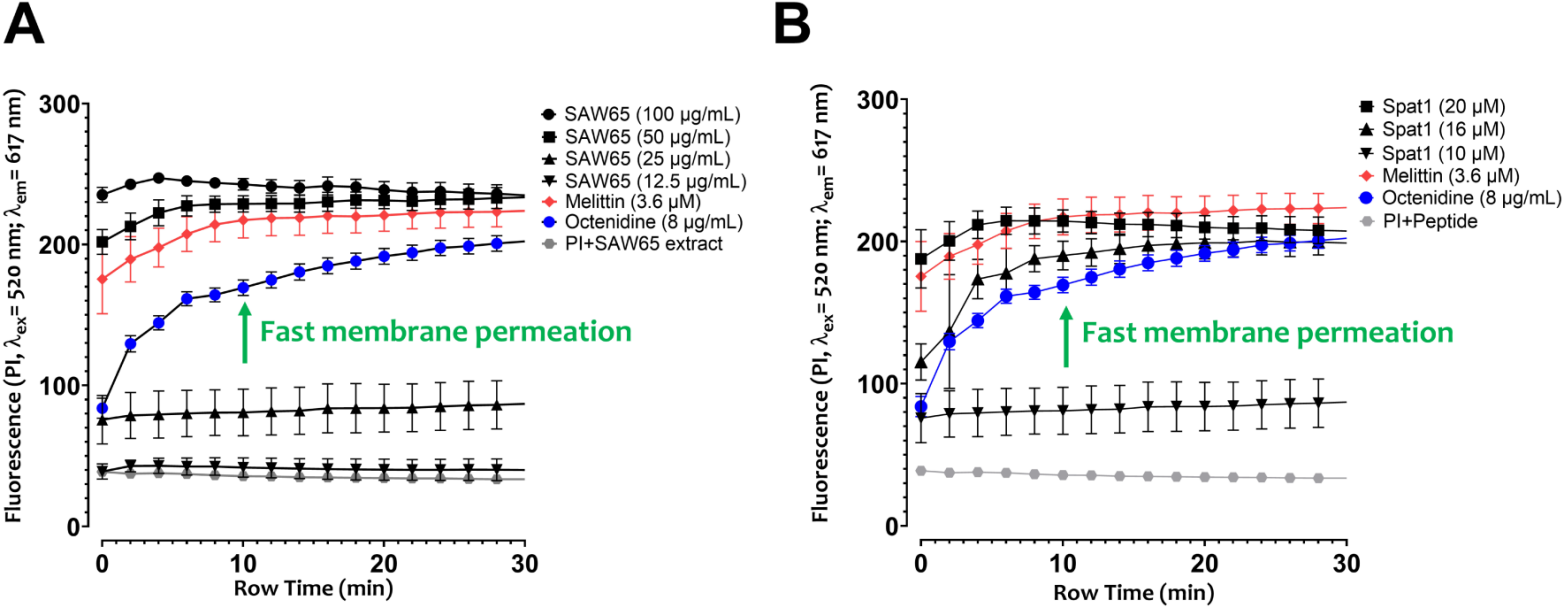
Membrane activity assays of peptide extract and purified Spat1. Permeabilization of bacterial membranes by peptide extract SAW65 (A) and Spat1 (B). *B. subtilis* was labeled with PI and exposed to different concentrations of Spat1 (sub-LC_99.9_ and LC_99.9_), as well as sub-LC_99.9_, LC_99.9_, and above LC_99.9_ of SAW65. The influx of PI was monitored by measuring PI fluorescence using fluorometry at a duration of 2 h (graphs show only the first 30 min after exposure to peptides). The results are shown as the means (and standard deviation) of three independent experiments.

### Biochemical Derivatization and *De Novo* Peptide Sequencing of the Cyclotide Spat1

Reduction and alkylation of Spat1 induced a + 348 Da mass shift. Subsequent EndoGluC digestion yielded an additional +18 Da shift (total = +366 Da) (Figure 5A), confirming cleavage at a single glutamic acid residue. MALDI-TOF MS/MS of the EndoGluC digest revealed Spat1’s linear sequence as NH_2_–SCVYL/IPCFTSVL/IGCSCSNKVCYKNGL/IPCGE–COOH (Figure 5B). Tryptic digestion of carbamidomethylated Spat1 produced two fragments: [M + H]^+^ 2966.11 Da (NH_2_–NGL/IPCGESCVYL/IPCFTSVL/IGCSCSNK–COOH; Figure 5C) and 569.27 Da (NH_2_–VCYK–COOH; Figure 5D), indicating C-terminal hydrolysis to two basic residues. Chymotryptic digestion yielded fragments at [M + H]^+^ 2151.73 Da (NH_2_–L/IPCFTSVL/IGCSCSNKVCY–COOH) and 1383.60 Da (NH_2_–KNGL/IPCGESCVY–COOH; Figure S3, Supporting Information), demonstrating C-terminal cleavage to two aromatic/hydrophobic residues. Summary of fragmentation ions for EndoGluC and trypsin digests is presented in Tables S3–S5, Supporting Information.

**Figure 5:**
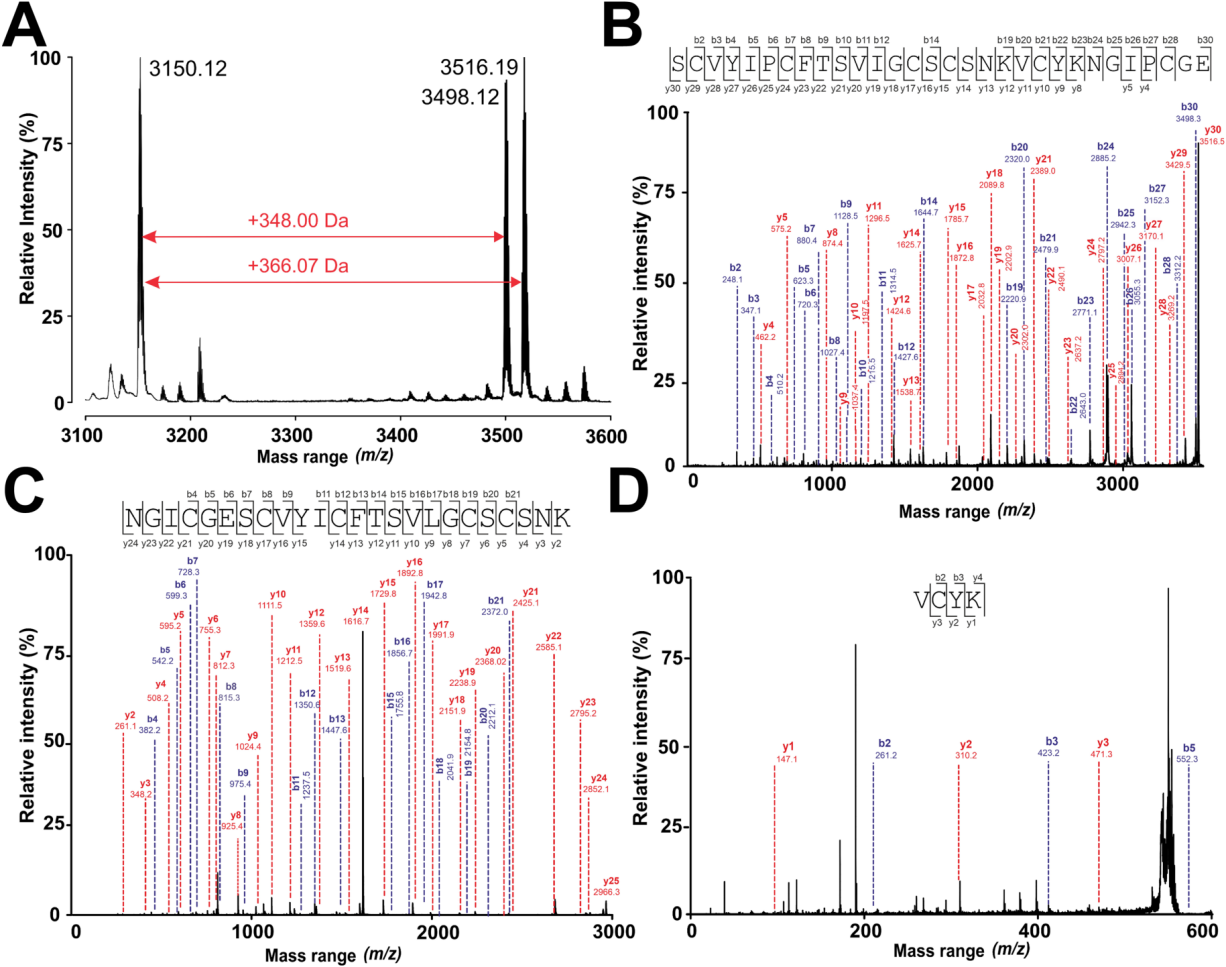
MALDI-TOF MS/MS de novo sequencing of Spat1. (A) Ring opening of the native peptide *m*/*z* 3150.12 to a linear product *m*/*z* 3516.19 was observed in a digestion experiment with endopeptidase GluC. The *m*/*z* 3496.12 corresponds to the cyclic reduced and S-acetamidated intermediate. (B) The peptide *m*/*z* 3516.19 was used for an MS/MS fragmentation experiment, and the b- and y-ions of the received fragmentation spectrum were de novo annotated, providing the full-length amino acid sequence of Spat1. Similarly, a trypsin digestion experiment was carried out, and two tryptic fragments, *m*/*z* 2966.3 (C) and *m*/*z* 570.3 (D), were observed. The annotated fragmentation spectra further confirmed the primary amino acid sequence of Spat1. Note: the isobaric amino acids leucine and isoleucine were confirmed by a separate chymotrypsin digestion experiment (Figure S3, Supporting Information), and high-sensitivity amino acid analysis (Table S6, Supporting Information). All mass signals are presented as monoisotopic masses [M + H]^+^.

The isobaric leucine and isoleucine were analyzed by high-sensitivity amino acid (hsAA) analysis of the Spat1, confirming the presence of isoleucine rather than leucine (Table S6, Supporting Information). After assembling the observed consistent sequences in all enzymatic digests, and the amino acid composition of the hsAA analysis compared to the theoretical calculation, the primary sequence of the Spat1 peptide was determined as cyclo-GIPCGESCVYIPCFTSVIGCSCSNKVCYKN.

A UniProt BLAST search for Spat1 returned approximately 150 hits. Sequences with ≥75% identity (top 45 hits; Table S7, Supporting Information) were selected for multiple sequence alignment (Figure 6). Phylogenetic analysis reveals Spat1 forms a sister branch to the kalata B5-hyen-E monophyletic clade, suggesting a shared ancestral origin but sufficient sequence divergence to constitute a distinct lineage (Figure 6). This pattern is consistent with scenarios such as an early divergence, a lineage-specific gene duplication, or accelerated evolution in Spat1’s lineage. Rubiaceae and Loganiaceae are both in the order Gentianales and are therefore more closely related to each other than either is to Violaceae, which belongs to the distant order Malpighiales. Hence, Loganiaceae’s Spat1 is not expected to sit near a clade containing both Rubiaceae and Violaceae using organismal (species-level) phylogeny alone. Such a pattern is therefore more consistent with gene-level processes like ancient duplication and differential retention, convergent sequence evolution under shared functional constraints, or rate variation, rather than simple inheritance that mirrors plant family relationships^13,34,35^ (Figure 6).

**Figure 6:**
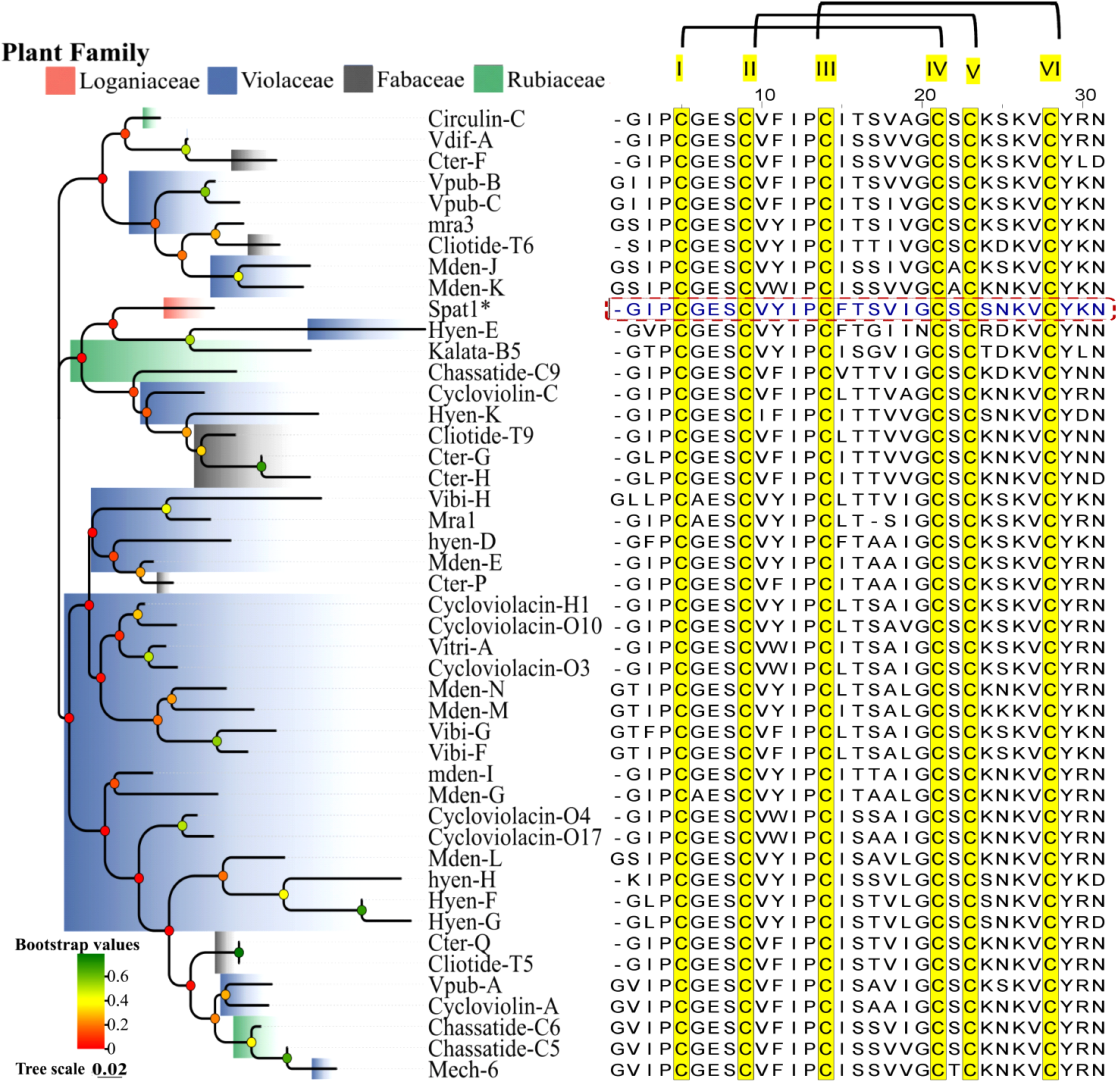
Phylogenetic relationship of Spat1 (asterisk; dashed rectangular) with other cyclotides (≤86% similarity) from the UniProt BLAST interface. Using a neighbor-joining tree with bootstrap consensus values (0 to 1) derived from 1000 replicates, together with a multiple sequence alignment of the disulfide linkage pattern, Spat1 displayed a monophyletic separation from other known cyclotides, affirming its sequence novelty.

### Three-Dimensional Structure Prediction, Comparison, and Electrostatic Characterization of Spat1

Visualization of the AlphaFold-predicted Spat1 structure, using AFCycDesign cyclic peptide modeling (per residue prediction confidence ≥ 90%; Figure S4, Supporting Information) in ChimeraX, revealed a cystine-knot fold interconnected by six loops, containing three β-sheets and a 3_10_-helix, (Figures 7A and 7B). Surface representation further illustrates the hydrophobic spatial distribution of individual amino acids across the peptide (Figure 7C). Despite sequence variations in loops 3 and 5, the structural alignment between Spat1 and cyO2 exhibits minimal differences in their secondary structures, as evidenced by an RMSD of 0.648 Å (Figure 7D). Further, Spat1 contains three charged residues: two lysines (K) and one glutamic acid (E) (Figure 7E). The PYMOL calculation predicted its net surface charge at pH 7.0–7.4 to be +1. In contrast, cyO2 possesses four charged residues: two lysines (K), one arginine (R), and one glutamic acid (E) (Figure 7F). PYMOL analysis yielded a net charge of +2 at physiological pH (7.0–7.4), consistent with experimental data.^17^

**Figure 7:**
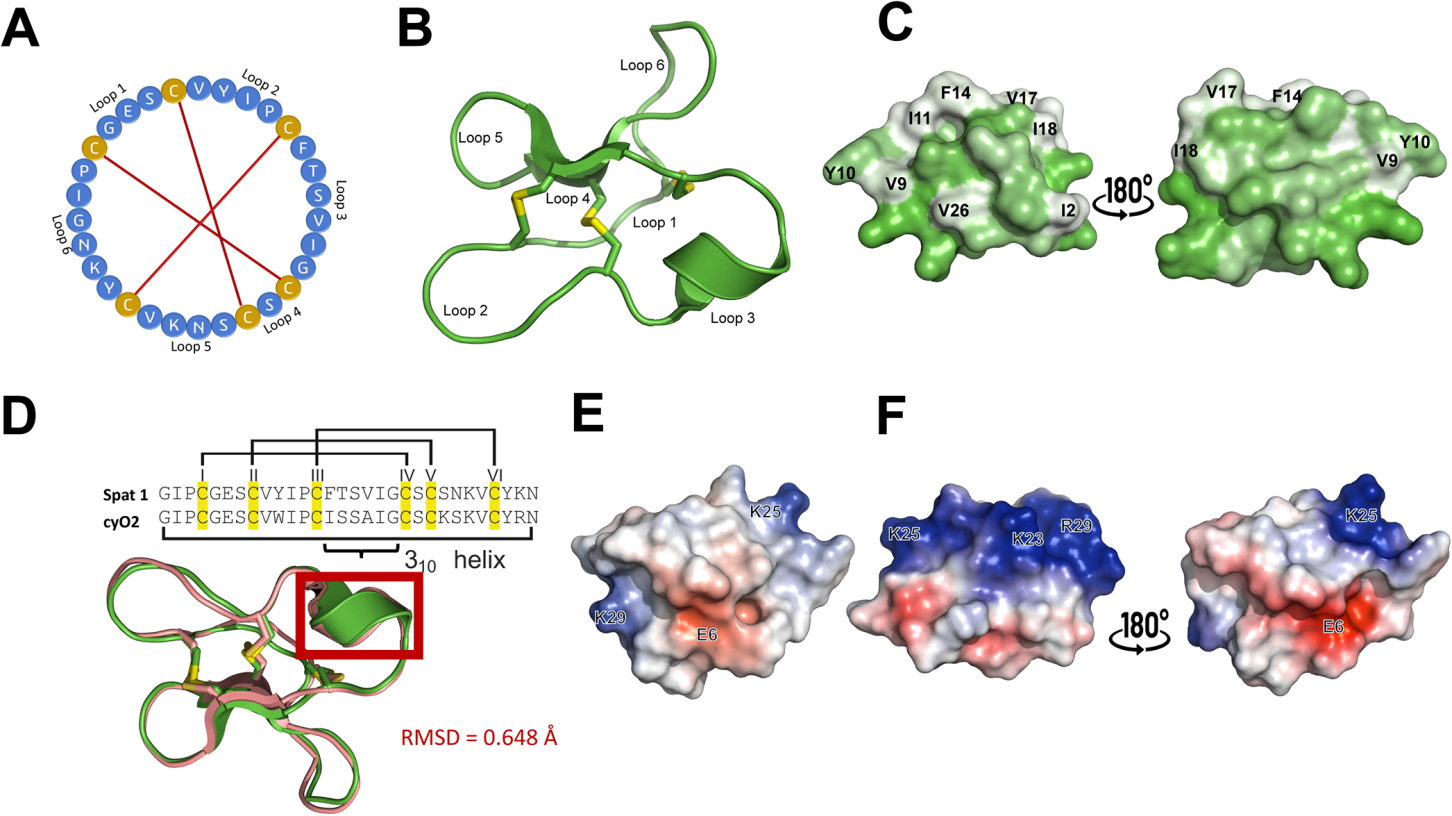
Structural representations of cyclic Spat1. Primary sequence in wheel representation (A), cartoon representation displaying the two antiparallel β-sheets, the 3_10_-helix, and three disulfide linkages (yellow) (B); intercysteine loops are indicated. Surface representation showing the hydrophobicity (C) of Spat1 using the Eisenberg hydrophobicity scale;^36^ hydrophobic residues are displayed in green, whereas hydrophilic residues are displayed in white. Structural alignment of Spat1 (green) and the bracelet cyclotide cycloviolacin O2 (salmon) at RMSD = 0.648 Å (D); sequence alignment is shown above the structure with disulfide bond connectivity outlined. Electrostatic potential of Spat1 (E) and cyO2 (F) outlining negatively charged residues in red and positively charged residues in blue.

### Bactericidal Mode-of-Action of Spat1

Spat1 exhibits antibacterial activity that is comparable to other known antibacterial cyclotides. Notably, as a bracelet subfamily cyclotide, Spat1 shares this functional characteristic with most bracelet-type cyclotides such as cyO2, cter G, cter R, kB7, cyI3–cyI6, contrasting with the typically inactive/less active Möbius subfamily.^17,20,37–42^

Plant AMPs, including the cyclotide family, exhibit substantial diversity. This arises from amino acid variations in noncysteine residues within the conserved cystine-knot framework, enabling diverse functions. Surface charge, hydrophobicity, polarity, and 3D conformation primarily govern functional differences across families.^43^ Cyclotides primarily exert antimicrobial effects via membrane disruption, facilitated by their amphipathic structure and interactions with microbial membranes.^44,45^ Spat1 readily penetrated the cytoplasmic membrane of Gram-positive *B. subtilis* despite low interaction with the most anionic component of the *Bacillus* envelope-LTA. Spat1 showed very low LTA binding affinity (<20% of octenidine, which has a comparable overall net charge of two positively charged residues) as well as comparable killing capacity of *B. subtilis* devoid of LTA. Of note, LTA binding was assessed using BC-cadaverine, which binds specifically to the anionic regions of lipoteichoic acid, suggesting that interaction with teichoic acid chains is also unlikely. Interaction with wall teichoic acid – the nonlipophilic counterpart of lipoteichoic acid in Gram-positive bacteria – can similarly be ruled out. These findings clearly indicate that the peptide’s activity does not depend on interactions with either lipoteichoic acid or wall teichoic acid, nor is its membrane penetration affected by them. Gram-positive *B. subtilis* and *S. aureus* majorly differ by the presence of zwitterionic PE in *B. subtilis*.^46^ More so, the high concentration of L-PG in *S. aureus* significantly reduces the net negative charge of its membrane compared to *B. subtilis* (which relies more on PG and CL). The positively charged lysine on L-PG electrostatically repels positively charged cationic AMPs (e.g., defensins) and reduces their binding/insertion into the membrane. *B. subtilis* lacks this major mechanism, making it relatively susceptible to Spat1.^47,48^ Conversely, Spat1, within an hour of incubation, failed to penetrate the *E. coli* membrane, likely due to the lipopolysaccharide (LPS)-containing outer membrane of Gram-negative bacteria, which usually presents a structural obstacle that might hinder entry and limit access to the potential target site. This is in contrast to the established activity of *Viola odorata’s* cyO2 against Gram-negative bacteria.^17^ The studies by Henriques et al.^44^ demonstrated that cyclotides with a net charge below +2 generally lack activity against *E.  coli*. For instance, kalata B1 (kB1) fails to bind and disrupt negatively charged membranes, including those containing LPS.^45^ A similar limitation may apply to Spat1, which has a net charge of +1. In general, for highly cationic peptides such as SAAP-148 and OP-145, neutralization of the bacterial surface is more important for antimicrobial activity against Gram-negative bacteria than Gram-positive. In Gram-positive bacteria like *E.  hirae*, the more porous cell wall allows peptides easy access to the cytoplasmic membrane, whereas in Gram-negative bacteria such as *E. coli*, the additional LPS layer prevent direct access, making surface charge neutralization a critical step.^49^ We also cannot exclude the possibility that prolonged incubation with *E. coli* might improve activity; however, since higher concentrations did not enhance the effect, it is less likely that the peptide exhibits activity against *E. coli* within the same concentration range as observed for Bacillus. This discrepancy may stem from electrostatic factors. Electrostatic forces significantly impact Gram-negative bacterial surfaces more than their cytoplasmic membranes.^17,49^ For AMPs like cyclotides, net charge and hydrophobic residue distribution critically affect interactions with negatively charged LPS on the outer membrane of Gram-negative bacteria (e.g., *E. coli*).^50^ Positively charged residues promote LPS binding and membrane penetration.^51–54^ Spat1’s inability to penetrate *E. coli*, unlike cyO2, likely relates to its lower net charge (+1 vs +2 for cyO2), determined by charged residues (K, R, D, E; Figures 7D–7F). This charge difference might give cyO2 a penetrative advantage. Electrostatic interactions are essential for Gram-negative penetration: blocking charged residues in cyO2 reduced its anti-*Salmonella* activity,^17^ and synthetic peptides OP-145 and SAAP-148 rely on similar interactions.^49,55,56^

Cyclotides typically exhibit limited or no direct inhibitory activity against *S. aureus*, as reported extensively.^17,39,42^ Although *in vivo* data suggest cyO2 and kalata B2 may reduce bacterial load and enhance phagocytosis,^39^ this may point to indirect immunomodulatory effects rather than direct bactericidal action. Consistent with this trend, Spat1 also failed to inhibit *S. aureus* despite targeting Gram-positive bacteria. This widespread cyclotide inefficacy against *S. aureus* is likely attributable to its lack of PE – a key lipid target for cyclotide membrane disruption.^37,44,57^ Recent structural work reveals ionic interactions between a conserved loop 1 glutamic acid (E) in cyclotides and the PE headgroup ammonium ion.^57^ Thus, Spat1’s E6 residue may enable selective recognition and binding to PE-containing membranes (e.g., in *B. subtilis*) via ionic attraction, further stabilized by hydrogen bonding, cation-π, and CH−π interactions.

While PE binding is necessary for cyclotide antimicrobial activity, it is insufficient alone, as not all cyclotides bearing the conserved glutamic acid exhibit potent effects. Hydrophobicity is a critical factor.^47,48^ Spat1 and cyO2 feature a 3_10_-helix in loop 3, typically amphipathic with distinct hydrophobic and hydrophilic faces. This amphipathicity facilitates membrane binding: the hydrophilic side associates with aqueous solvent or lipid head groups, while the hydrophobic face embeds into the membrane core. Research confirms that α-helical amphiphilicity in AMPs is pivotal for antimicrobial action, enabling membrane penetration and destabilization.^58,59^ For instance, enhancing AMP amphiphilicity boosts bactericidal activity via stronger hydrophobic membrane interactions,^50^ while disrupting α-helices reduces efficacy.^60^ Also, hydrophobic patch location influences cyclotide binding orientation and membrane penetration.^61^ They share hydrophobic patches over loops 2 and 3 (Figure 7C), potentially enhancing penetration into bacterial membrane bilayers. Their structural similarity and conserved hydrophobic topology may contribute to their activity against Gram-positive *B. subtilis*. The membrane interaction scheme of Spat1 is summarized in Figure S5, Supporting Information.

## Conclusions

Cyclotide distribution across plant phylogeny appears to be a result of convergent evolution, with many plant families still unexplored and potentially harboring novel cyclotides with valuable bioactivities. Spat1, identified here for the first time in *Spigelia anthelmia* (Loganiaceae), represents a new cyclotide with bactericidal activity against Gram-positive *Bacillus subtilis* through membrane disruption, most likely mediated by PE interaction. However, further studies are needed to confirm the involvement of PE in the mode of action of Spat1. To address Spat1’s limited activity against Gram-negative bacteria, future studies should focus on structure–activity relationship analyses aimed at enhancing membrane permeability, potentially by increasing the number of basic residues to improve lipopolysaccharide binding. Expanding cyclotide screening across unexamined plant families is essential. A deeper understanding of the convergent evolutionary forces shaping cyclotide distribution will not only facilitate predictive discovery of therapeutically relevant peptides but also shed more light on their ecological/biological functions.

## Experimental Section

### General Experimental Procedures

Plant peptide analysis and purification were performed using RP-HPLC, which exploits the amphipathic properties of peptides for effective separation. For SPE we used Phenomenex C_18_ cartridges (Aschaffenburg, Germany) with buffers: A: 99.9% ddH_2_O, 0.1% (v/v) TFA; B: 90% (v/v) acetonitrile, 0.1% (v/v) TFA in ddH_2_O. Analytical RP-HPLC employed Phenomenex Jupiter (150 × 2 mm, 5 μm, 300 Å) and Kromasil (250 × 4.6 mm, 5 μm, 100 Å) C_18_ columns. Linear gradients (5–80% B) flowed at 1 mL/min or 0.3 mL/min using A/B mobile phases. Preparative HPLC: Phenomenex column (300 Å, 250 × 21.2 mm, 10 μm; 8 mL/min). Semipreparative HPLC: Phenomenex column (300 Å, 250 × 10 mm, 10 μm; 4 mL/min). Fractionation was performed using a Dionex 3000 LC (Amsterdam, Netherlands). MALDI-TOF/TOF MS (ABSciex 4800, Framingham, MA) analyzed peptides in reflector-positive mode (3500 laser intensity; 2000–10000 shots). Samples (0.5 μL peptide + 3 μL α-CHCA) were spotted (0.5 μL) on 384-target plates. Data Explorer Software (ABSciex) processed spectra.

### Plant Selection, Collection, Authentication, and Preparation

Cysteine-rich peptide screening was conducted across selected members of three plant families. Specimens included: whole plants of *Euphorbia hirta*, *E. graminea*, and *E. hyssopifolia* (Euphorbiaceae); leaves and roots of *Anthocleista vogelii*, *A. djalonensis*, and *A. liebrechtsiana* (Gentianaceae); whole plant of *Spigelia anthelmia* and leaves of *Strychnos spinosa*, *S. floribunda*, and *S. inocua* (Loganiaceae). Plant materials were collected from the University of Ibadan campus surroundings and Ipara, Ogun State (notably for *Spigelia* and *Strychnos* species; 7°0′0″N, 30°40′0″E), and authenticated at the Forestry Herbarium Ibadan (FHI). Samples were air-dried, pulverized, and subjected to chemical extraction for downstream peptide analysis.

### Small-Scale Plant Cyclotide Screening

Cysteine-rich peptides were extracted using established protocols.^62–64^ Briefly, 1–2 g powdered plant material was extracted in 50 mL Falcon tubes with DCM/MeOH (1:1 v/v) for 16–20 h at 26–33 °C under mechanical agitation. Water addition generated aqueous-rich fractions by reducing methanol content. These fractions underwent RP-SPE: washing with 10% buffer B yielded SAW10 fractions, while 80% buffer B elution produced SAP80 (partially purified extracts). After lyophilization, extracts were analyzed via RP-HPLC (late-elution screening) and MALDI-TOF MS (2500–4000 Da detection). Analytical RP-HPLC used a Kromasil column (250 × 4.6 mm, 5 μm, 100 Å) with 5–65% buffer B gradient at 1 mL/min. Cysteine residues were detected via biochemical derivatization (DTT/iodoacetamide), while cyclic backbones were assessed by proteolytic degradation. Briefly, extracts or purified peptides (5 μg) in 0.1 M NH_4_HCO_3_ were reduced upon treatment with DTT (10 mM, pH 8.5; 60 °C, 3 h). Alkylation was done with IAA (50 mM final) added to the reduced samples (65 °C, 1 min). Quenching: DTT (10 mM) quenched the reaction (RT, 10 min). Digestion was done with EndoGluC (0.5 μg), trypsin, or chymotrypsin (0.4 μg each), cleaving peptides (37 °C, 3 h); TFA (3% final) halted digestion. Peptide masses were monitored by MALDI-TOF MS postreduction/alkylation/digestion per established protocols.

### Peptide Enrichment

Lyophilized partially purified extracts were dissolved in Buffer A and analyzed via analytical HPLC. Fractions collected at 10 min intervals were analyzed by MALDI-TOF MS to identify peptide-eluting regions. This informed SPE protocol optimization: aqueous-rich fractions were processed by RP-SPE with a 30% buffer B wash (yielding SAW30) and 65% buffer B elution (yielding SAP65). Both fractions were reanalyzed by MALDI-TOF MS to verify retention of all initial extract peptide peaks. Finally, reverse-phase analytical HPLC profiles of peptide-rich fractions were acquired.

Lyophilized extracts were dissolved in buffer A and subjected to analytical HPLC. Fractions were collected and analyzed on MALDI-TOF MS to ascertain the peptide-eluting region. Hence, the protocol for SPE was optimized based on the peptide-eluting region from the analytical RP-HPLC profile. For enrichment, the aqueous-rich fractions were resubjected to RP-SPE, and peptides were eluted with 65% buffer B after washing with 30% buffer B to obtain SAP65 and SAP30, respectively. SAP30 and SAP65 were again subjected to MALDI-TOF MS to confirm the presence of all peptide peaks present in the initial partially purified extracts. Thereafter, the HPLC analytical profiles of the peptide-rich fractions were obtained in reverse phase mode.

### Large-Scale Peptide Extraction, Isolation, and Characterization

To enable comprehensive peptide isolation, substantial quantities of plant material were harvested. The optimized extraction protocol (detailed earlier) was applied to powdered samples, beginning with large-scale solvent extraction: 100 g of dried plant powder was combined with one liter of a 1:1 methanol-dichloromethane mixture and stirred continuously overnight at room temperature. After filtration to remove plant debris, the filtrate underwent aqueous partitioning by adding half its volume of deionized water, separating the peptide-rich aqueous-methanol phase from nonpolar components. This aqueous layer was concentrated using a Heidolph Hei-VAP vacuum evaporator and freeze-dried to yield the crude aqueous extract (designated Aq). This entire extraction sequence was repeated across five independent batches using fresh starting material. The combined freeze-dried Aq extracts were dissolved in buffer A and loaded onto a C_18_ ZEOprep 60 Å solid-phase extraction cartridge (40–64 μm particle size; Zeochem, Switzerland), pre-equilibrated with buffer A. Following a wash step with 30% buffer B to remove impurities, peptide-rich fractions were eluted using 65% buffer B, generating samples designated SAW65 and RVL65. All five SPE batches were quality-controlled via MALDI-TOF MS to confirm peptide retention. SAW65 underwent bioactivity-guided fractionation. Briefly, the freeze-dried material was reconstituted in 5% buffer B and loaded onto a preparative HPLC column. Nine fractions were collected automatically at 5 min intervals across a 30% to 75% buffer B gradient, with peptide elution monitored by UV absorbance at 214, 254, and 280 nm. Further purification on a semipreparative column yielded a homogeneous peptide isolate. This purified peptide was treated with reduction-alkylation and enzymatic digestion (as per the previously described method). Following derivatization, it was subjected to proteolysis using EndoGluC, trypsin, and chymotrypsin. The resulting peptide fragments were individually analyzed via tandem MS. Spectra were interpreted by manually reconstructing amino acid sequences through alignment of identified N-terminal (b-ions) and C-terminal (y-ions) fragment series.

### High-Sensitivity Amino Acid Analysis

To conclusively verify the amino acid sequences derived from tandem MS and resolve isobaric residues like leucine/isoleucine, high-sensitivity amino acid analysis was conducted via commercial service provider Australian Proteome Analysis Facility (Sydney, Australia) using HPLC precolumn derivatization. Samples were gently mixed for 20 min in 0.1% TFA/Milli-Q water. Duplicate aliquots were dried and subjected to 24-h gas-phase hydrolysis in 6 M HCl at 110 °C. Under these conditions, asparagine converts to aspartic acid, while glutamine converts to glutamic acid (reported values for Asp/Glu represented totals of both forms). Cysteine and tryptophan are not analyzable by this method. Hydrolyzed amino acids were then tagged via Waters AccQTag Ultra chemistry according to manufacturer protocols and separated/quantified on a Waters Acquity UPLC system. All samples underwent duplicate runs, with results expressed as mean values.

### Sequence Homology and Phylogenetic Analysis

Following confirmation of amino acid sequences through mass spectrometry and high-sensitivity analyses, we performed database searches to identify homologous peptides. Mature sequences of isolated peptides served as queries in UniProt’s BLASTp interface, supplemented by literature mining, to retrieve reference sequences (RefSeq) and reveal significant sequence similarities. Subsequent multiple sequence alignment was executed using Clustal Omega (v1.2.4) via UniProt, with visualizations generated in JalView (v2.11.4.1). Phylogenetic relationships were then reconstructed using the Neighbor-Joining algorithm in MEGA11 and visualized on One Table (tvBOT).^65,66^

### Peptide Three-Dimensional Structural Analysis

Spat1 peptide tertiary structure was predicted via AlphaFold,^67^ leveraging its unprecedented accuracy in biomolecular structure modeling to elucidate functional interactions, in combination with the cyclic peptides workflow for structure prediction and design (AfCycDesign), engaging a cyclic offset matrix.^68^ Structural analyses – including molecular visualization, superposition, electrostatic potential mapping, and disulfide bond assignment to confirm knotted topology – were performed using UCSF ChimeraX v1.10 ^69^ and PYMOL 3.1.1.^70^ The structure assignment program STRIDE was used to confirm the predicted secondary structures.^71^

### Bactericidal Assay

Antimicrobial efficacy of peptides and partially purified fractions was evaluated per established protocols.^47^ Overnight cultures of *E. coli*, *B. subtilis* (wild-type and *B. subtilis* AK066B *yfnI*::*erm yqgS*::*spc ltaS*::*cat* strain depleted of lipoteichoic acid, ΔLTA mutant),^32^ and *S. aureus* ATCC25923 were prepared from single colonies in Mueller-Hinton broth (MHB CarlRoth; 37 °C, 200 rpm). Main cultures were inoculated at OD_600_ = 0.05 and grown to mid log phase (MHB, 37 °C, 200 rpm). After 3.5 h, cells were washed once with sodium phosphate buffer (PBS, 130 mM NaCl, 20 mM NaH_2_PO_4_/Na_2_HPO_4_, pH 7.4). Bacterial suspensions (1 × 10^6^ CFU/mL in PBS, OD_600_-verified) were incubated with peptides for 1 h (37 °C, 200 rpm). The peptide concentration range was tested between 10 μM and 20 μM. Viability was assessed by plating 100 μL of 10-fold serial dilutions on Mueller–Hinton agar (Carl Roth), with colonies enumerated after overnight incubation. Bactericidal activity was quantified as lethal concentration LC_99.9_: the lowest concentration that killed ≥99.9% of the initial population (≤10 colonies).^47,49,72^

### Bacterial Membrane Permeabilization Assay

To assess peptide membranotropic activity in susceptible bacteria, we employed propidium iodide (PI) – a membrane-impermeant fluorescent DNA stain that only enters cells with compromised membranes. Mid log phase *B. subtilis* was washed twice with PBS and adjusted to 1 × 10^7^ CFU/mL. In foil-wrapped black 96-well plates, 10 μL of peptide-rich fractions or pure peptides were incubated with bacterial suspensions (final density: 1 × 10^6^ CFU/mL) and 5 μL PI (50 μg/mL). Fluorescence kinetics (excitation: 520 nm; emission: 617 nm) were monitored for 2 h at 37 °C using a Promega GloMax-Multi+ Detection System (USA). The percentage PI uptake was measured as $\%PI uptake=100\%\times \left(P_{x}-P_{o}\right)÷(P_{100}-P_{o}),$ , where *P*_x_ = PI + cells in the presence of peptides; *P*_o_ = PI + cells alone in PBS buffer; *P*_100_ = 100% PI + cells (determined from Triton-X-100). Melittin and octenidine, with well-demonstrated membrane permeabilization effects, were used as positive controls.^73^

### Statistical Analysis

Experiments were done in triplicate (biological *n* = 3), and data were analyzed and plotted at different time intervals in Microsoft Excel and GraphPad Prism 8 (GraphPad Software, San Diego), respectively. Data were expressed as mean ± SD.

## Supplementary Material



## Notes

Additional experimental methods, including the BODIPY-cadaverine (lipoteichoic acid displacement) assay (*Additional Methods*); MALDI-TOF mass spectrometry of plant samples (*Figure S1*); LTA-binding potential of Spat1 (*Figure S2*); MALDI-TOF MS/MS de novo sequencing of chymotrypsin-digested Spat1 (*Figure S3*); small-scale cyclotide screening in plant samples (*Table S1*); bactericidal effects of peptide extracts and fractions against *E. coli* and *B. subtilis* (*Table S2*); *de novo* annotation of EndoGluC and trypsin fragment ions of Spat1 (*Tables S3–S5*); high-sensitivity amino acid and residue analysis of Spat1 (*Table S6*); sequence similarity of Spat1 to UniProt bracelet cyclotides (*Table S7*); cyclic peptide structure prediction using AFCycDesign (*Figure S4*); and a proposed membrane interaction scheme of Spat1 (*Figure S5*) (PDF)
