# Supplementary material for: Discovery, Isolation, and Bactericidal Activity of a Cyclotide from Spigelia anthelmia L. (Loganiaceae)
Source: J Nat Prod. 2026 Jan 12;89(1):139–50. doi: 10.1021/acs.jnatprod.5c01216 (PMC12836362; doi:10.1021/acs.jnatprod.5c01216)
Supplement: Supplementary file 1 [file np5c01216_si_001.pdf]

Supporting Information

Discovery, isolation and bactericidal activity of a cyclotide from *Spigelia anthelmia* L. (Loganiaceae)

Toluwanimi E. Akinleye[a,b,c], Latifat O. Sidiq[b,d], Alfred Attah[e], Roland Hellinger[a], Lisa Pabi[c], Nermina Malanovic[c,f,g]*****, Omonike O. Ogbole[b]*****, Christian W. Gruber[a]*****

[a]Center for Physiology and Pharmacology, Medical University of Vienna, Schwarzspanierstr. 17, 1090 Vienna, Austria.
[b]Department of Pharmacognosy and Herbal Medicine, Faculty of Pharmaceutical Sciences, University of Ibadan, 200284, Nigeria.
[c]Institute of Molecular Biosciences, University of Graz, Humboldtstraße 50/III, 8010 Graz, Austria.
[d]Department of Plant and Environmental Biology, Kwara State University, 241103, Nigeria.
[e]Department of Pharmacognosy and Drug Development, Faculty of Pharmaceutical Sciences, University of Ilorin, 240003, Nigeria.
[f]Field of Excellence BioHealth, University of Graz, 8010 Graz, Austria.
[g]BioTechMed Graz, 8010 Graz, Austria.

*****Corresponding authors: christian.w.gruber@meduniwien.ac.at; nikeoa@yahoo.com; nermina.malanovic@uni-graz.at

Contents

		page
**Additional Methods**	BODIPY-cadavarine assay (LTA acid displacement assay)	3
**Figure S1**	Mass spectrometry of plant samples	4
**Figure S2**	LTA binding potential of Spat1	5
**Figure S3**	MALDI-TOF MS/MS *de novo* sequencing of chymotrypsin-digested Spat1	6
**Table S1**	Small-scale cyclotide screening in plant samples	7
**Table S2**	Bactericidal effects of peptide extracts and fractions on Gram-negative *E. coli* and Gram-positive *B. subtilis*	8
**Table S3**	*De novo* annotation of the Spat1 EndoGluC fragment ions, m/z 3516.5	9
**Table S4**	*De novo* annotation of the Spat1 trypsin fragment ions, m/z 2966.5	10
**Table S5**	*De novo* annotation of the Spat1 trypsin fragment ions, m/z 570.3	11
**Table S6**	High-sensitivity amino acid and residue analysis of Spat1	12
**Table S7**	Similarity of Spat1 amino acid sequence to other UniProt bracelet cyclotides (BLASTp)	13
**Figure S4**	Prediction score of Spat1 using AFCycDesign cyclic peptide modelling	15
**Figure S5**	Proposed membrane interaction scheme of Spat1	16

Additional Methods

BODIPY-cadaverine assay (LTA acid displacement assay): LTA-binding affinity of the active peptide was quantified via competitive displacement of BODIPY-cadaverine (BC; Invitrogen ThermoFisher), a fluorescent probe [1,2]. This fluorescence-based assay measures binding strength through displacement efficiency: higher-affinity peptides release more BC into solution, increasing detectable fluorescence. In practice, BC (100 µg/mL methanol) and LTA (1 mg/mL in PBS buffer; Sigma Aldrich) were combined in black 96-well plates. Peptide solutions (10 µL) at varying concentrations were added to the mixture. Fluorescence intensity (excitation: 580 nm; emission: 617 nm) was recorded using a microplate reader. BC displacement percentage was calculated as: $\%\,BC\ displacement = F - Fo\ \div Fmax - Fo \times 100\%$ where Fo=fluorescence intensity at 617 nm at LTA saturation with BODIPY-cadaverine; Fmax= the fluorescence intensity without LTA.

Additional references

(1) Malanovic, N.; Leber, R.; Schmuck, M.; Kriechbaum, M.; Cordfunke, R. A.; Drijfhout, J. W.; de Breij, A.; Nibbering, P. H.; Kolb, D.; Lohner, K., Phospholipid-driven differences determine the action of the synthetic antimicrobial peptide OP-145 on Gram-positive bacterial and mammalian membrane model systems. Biochimica et Biophysica Acta (BBA) - Biomembranes, 2015, 1848(10, Part A), 2437-2447. DOI: https://doi.org/10.1016/j.bbamem.2015.07.010.

(2) Zorko, M.; Jerala, R., Alexidine and chlorhexidine bind to lipopolysaccharide and lipoteichoic acid and prevent cell activation by antibiotics. Journal of Antimicrobial Chemotherapy, 2008, 62(4), 730-737. DOI: 10.1093/jac/dkn270.

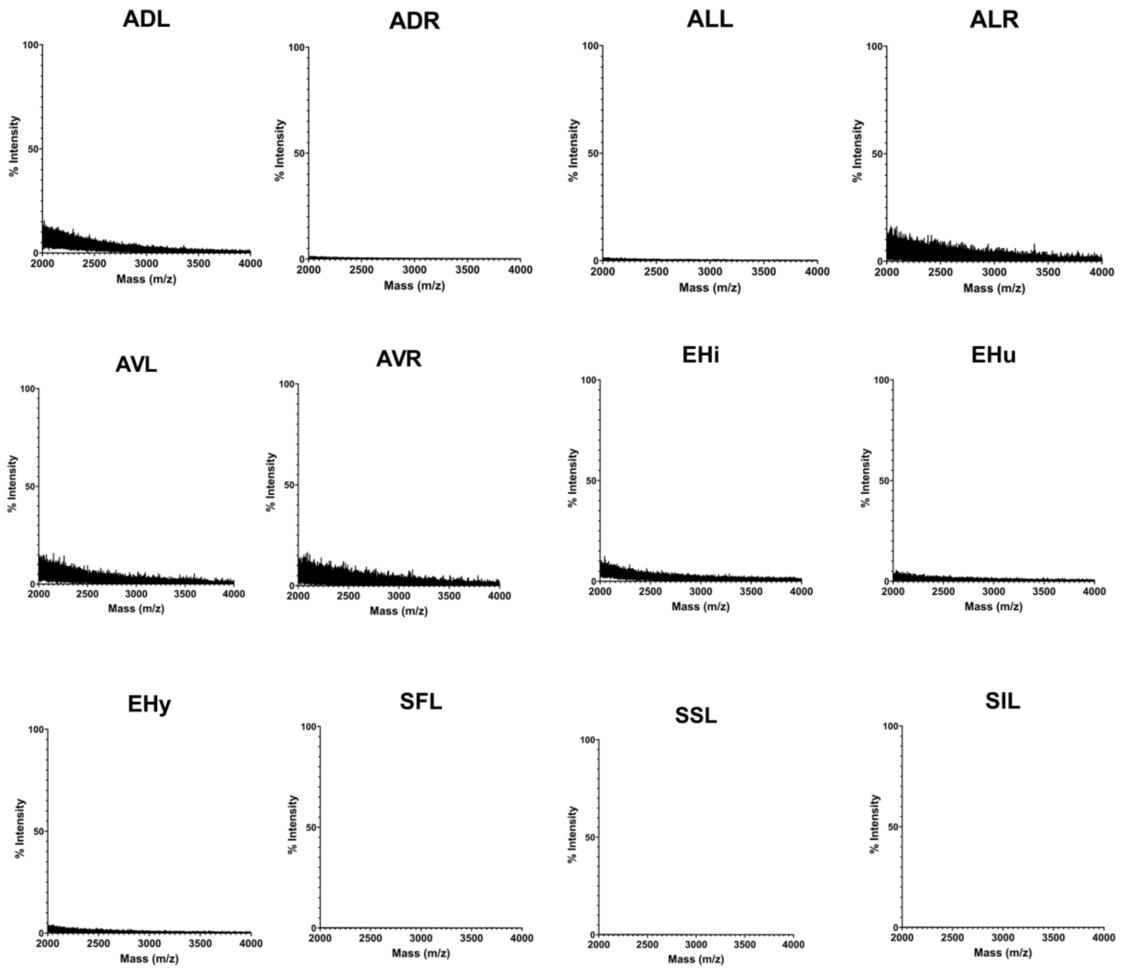

Figure S1. Mass spectrometry of plant samples. MALDI spectra of *Anthocleista djalonensis* leaf (ADL) and root (ADR), *A. liebrechtsiana* leaf (ALL) and root (ALR), *A. vogelii* leaf (AVL) and root (AVR); whole plant of *Euphorbia hirta* whole plant (EHi), *E. humifusa, E. hyssopifolia*; leaves of *Strychnos floribunda, S. inocua, S. spinosa*, showing no mass signal in the peptide mass range of 2000-4000 Da.

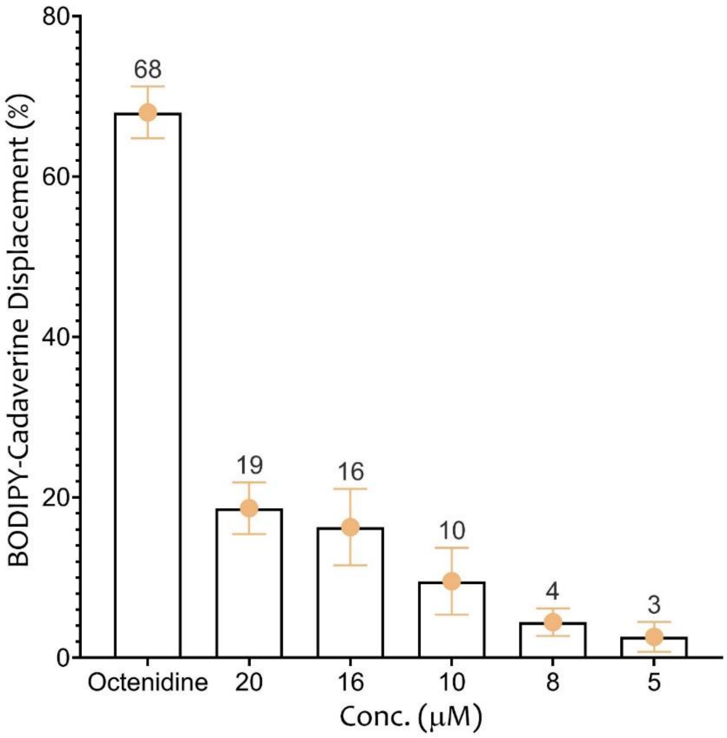

Figure S2. LTA binding potential of Spat1. LTA-binding affinity of the active peptide was quantified via competitive displacement of BODIPY-cadaverine (BC). The higher-affinity peptides should displace more BC, releasing it into solution. Spat1 showed low binding affinity for LTA, as it was unable to displace BC.

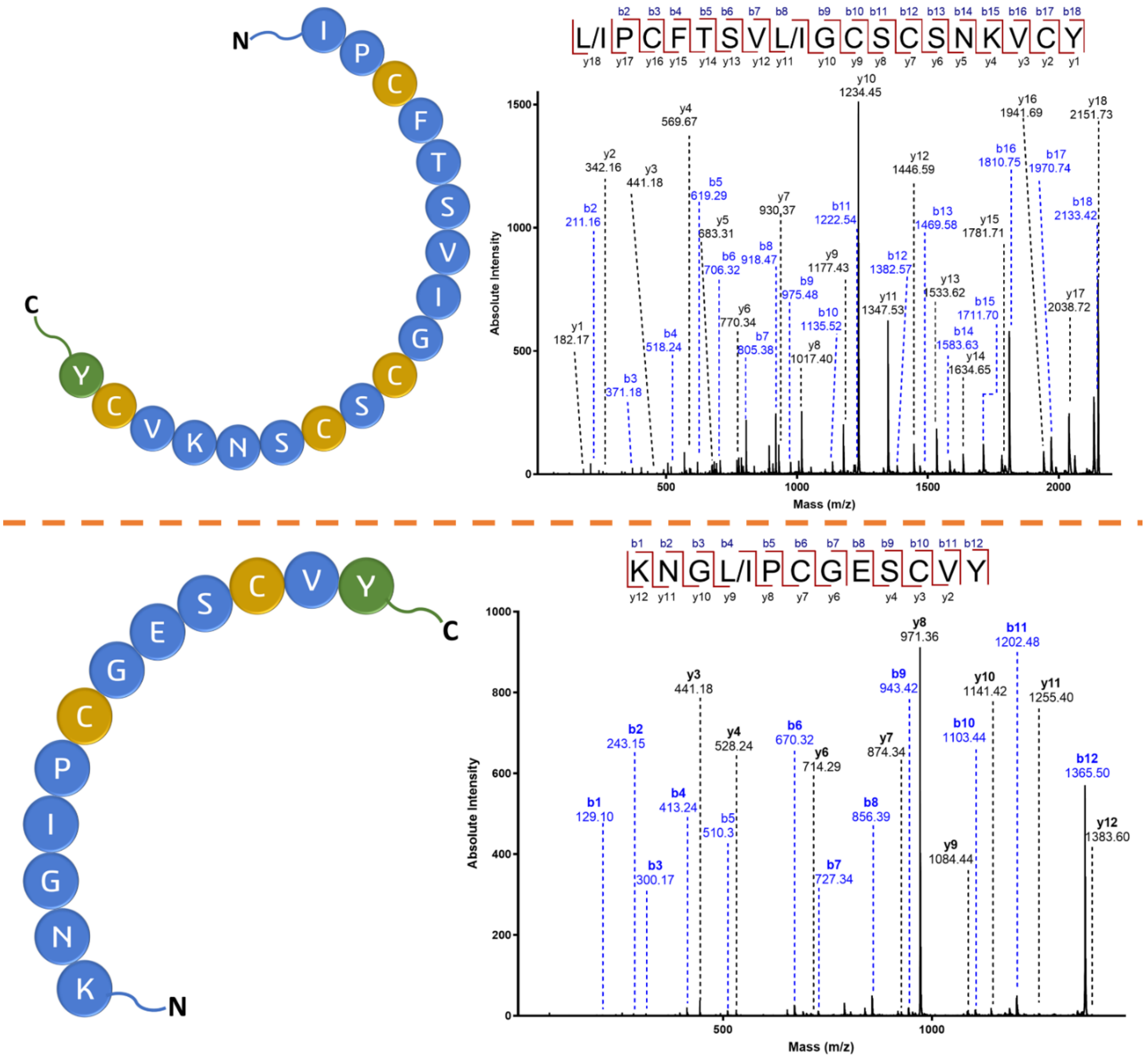

Figure S3. MALDI-TOF MS/MS *de novo* sequencing of chymotrypsin-digested Spat1. The b- and y-ions of two digested fragments were assigned following chymotryptic digestion and cleavage after the tyrosine C-terminus giving two chymotryptic fragments, m/z 2151.73 and m/z 1383.60. All mass signals are presented as monoisotopic masses $[M+H]^+$.

Table S1. Small-scale cyclotide screening in plant samples

S/N	Plant name	Plant family	Plant order	Part	Code	MALDI peptide signal(s)	Cysteine-rich	EndoGlu C digest
**1**	*Euphorbia hirta*	Euphorbiaceae	Malpighiales	Whole	EHiW	No	No	No
**2**	*Euphorbia graminea*	Euphorbiaceae	Malpighiales	Whole	EGW	No	No	No
**3**	*Euphorbia hyssopifolia*	Euphorbiaceae	Malpighiales	Whole	EHyW	No	No	No
**4**	*Spigelia anthelmia*	Loganiaceae	Gentianales	Whole	SAW	**Yes**	**Yes**	**Yes**
**5**	*Strychnos spinosa*	Loganiaceae	Gentianales	Leaf	SSL	No	No	No
**6**	*Strychnos floribunda*	Loganiaceae	Gentianales	Leaf	SFL	No	No	No
**7**	*Strychnos inocua*	Loganiaceae	Gentianales	Leaf	SIL	No	No	No
**8**	*Anthocleista djalonensis*	Gentianaceae	Gentianales	Leaf	ADL	No	No	No
**9**	*Anthocleista djalonensis*	Gentianaceae	Gentianales	Root	ADR	No	No	No
**10**	*Anthocleista liebrechtsiana*	Gentianaceae	Gentianales	Leaf	ALL	No	No	No
**11**	*Anthocleista liebrechtsiana*	Gentianaceae	Gentianales	Root	ALR	No	No	No
**12**	*Anthocleista vogelii*	Gentianaceae	Gentianales	Leaf	AVL	No	No	No
**13**	*Anthocleista vogelii*	Gentianaceae	Gentianales	Root	AVR	No	No	No

S/N, serial number

Table S2. Bactericidal effects of peptide extracts and fractions on Gram-negative *E. coli* and Gram-positive *B. subtilis*

**Sample**	**$LC_{99.9}$ (µg/mL)**	
	E. coli **(ATCC 25922)**	***B. subtilis*** **168**
SAW65	>1000	50.0 ± 0.0
SAW65 fraction D	>100	>100
SAW65 fraction E	>100	>100
SAW65 fraction F	>100	>100
SAW65 fraction G	>100	50 ± 0.0
SAW65 fraction H	>100	>100
vancomycin HCl	-	200.0 ± 0.0
octenidine HCl	2.0 ± 0.0	2.0 ± 0.0

Table S3. ***De novo*** **annotation of the Spat1 EndoGluC fragment ions, m/z 3516.5**

No.	Theor. Mass (m/z)	Mass deviation [ppm]	b-ion (m/z)	AA	y-ion (m/z)	Mass deviation [ppm]	Theor. Mass (m/z)	No.
1	88.039	n.a.	n.d.	**S**	3516.5	-19	3516.565	30
2	248.078	-89	248.1	**C**	3429.5	-10	3429.533	29
3	347.146	-11	347.15	**V**	3269.2	-90	3269.495	28
4	510.210	19	510.2	**Y**	3170.1	-103	3170.426	27
5	623.294	-10	623.3	**I**	3007.1	-88	3007.363	26
6	720.346	64	720.3	**P**	2894.2	-27	2894.279	25
7	880.385	-17	880.4	**C**	2797.2	-9	2797.226	24
8	1027.453	52	1027.4	**F**	2637.2	5	2637.188	23
9	1128.501	1	1128.5	**T**	2490.1	-8	2490.119	22
10	1215.533	27	1215.5	**S**	2389.0	-30	2389.072	21
11	1314.601	77	1314.5	**V**	2302.0	-17	2302.040	20
12	1427.685	60	1427.6	**I**	2202.9	-32	2202.971	19
13	1484.707	n.a.	n.d.	**G**	2089.8	-42	2089.887	18
14	1644.745	28	1644.7	**C**	2032.8	-32	2032.866	17
15	1731.777	n.a.	n.d.	**S**	1872.8	-15	1872.827	16
16	1891.816	n.a.	n.d.	**C**	1785.7	-53	1785.795	15
17	1978.848	n.a.	n.d.	**S**	1625.7	-35	1625.757	14
18	2092.891	n.a.	n.d.	**N**	1538.7	-16	1538.725	13
19	2220.986	39	2220.9	**K**	1424.6	-57	1424.682	12
20	2320.054	23	2320.0	**V**	1296.5	-67	1296.587	11
21	2480.093	78	2479.9	**C**	1197.5	-15	1197.518	10
22	2643.156	59	2643.0	**Y**	1037.4	-77	1037.480	9
23	2771.251	55	2771.1	**K**	874.4	-19	874.417	8
24	2885.294	33	2885.2	**N**	n.d.	n.a.	746.322	7
25	2942.315	5	2942.3	**G**	n.d.	n.a.	632.279	6
26	3055.400	33	3055.3	**I**	575.2	-100	575.257	5
27	3152.452	48	3152.3	**P**	462.2	58	462.173	4
28	3312.491	88	3312.2	**C**	n.d.	n.a.	365.120	3
29	3369.512	n.a.	n.d.	**G**	n.d.	n.a.	205.082	2
30	3498.555	73	3498.3	**E**	n.d.	n.a.	148.060	1

Table S4. ***De novo*** **annotation of the Spat1 trypsin fragment ions, m/z 2966.5**

No.	Theor. Mass (m/z)	Mass deviation [ppm]	b-ion (m/z)	AA	y-ion (m/z)	Mass deviation [ppm]	Theor. Mass (m/z)	No.
1	115.050	n.a.	n.d.	**N**	2966.3	0	2966.300	26
2	172.072	n.a.	n.d.	**G**	2852.1	n.a.	2852.257	25
3	285.156	n.a.	n.d.	**I**	2795.2	-13	2795.236	24
4	382.209	22	382.2	**P**	n.d.	n.a.	2682.152	23
5	542.247	87	542.2	**C**	2585.1	0	2585.099	22
6	599.268	-53	599.3	**G**	2425.1	16	2425.060	21
7	728.311	15	728.3	**E**	2368.02	-8	2368.039	20
8	815.343	53	815.3	**S**	2238.9	-43	2238.996	19
9	975.382	-19	975.4	**C**	2151.9	-30	2151.964	18
10	1074.450	n.a.	n.d.	**V**	1991.9	-13	1991.926	17
11	1237.513	11	1237.5	**Y**	1892.8	-30	1892.858	16
12	1350.597	n.a.	n.d.	**I**	1729.8	3	1729.794	15
13	1447.650	n.a.	1447.6	**P**	1616.7	-6	1616.710	14
14	1607.689	n.a.	n.d.	**C**	1519.6	-38	1519.657	13
15	1754.757	n.a.	1755.8	**F**	1359.6	-14	1359.619	12
16	1855.805	n.a.	1856.7	**T**	1212.5	-42	1212.550	11
17	1942.837	n.a.	1942.8	**S**	1111.5	-2	1111.503	10
18	2041.905	n.a.	2041.9	**V**	1024.4	-69	1024.471	9
19	2154.989	88	2154.8	**I**	925.4	-3	925.402	8
20	2212.011	-40	2212.1	**G**	812.3	-22	812.318	7
21	2372.049	21	2372	**C**	755.3	4	755.297	6
22	2459.081	n.a.	n.d.	**S**	595.2	-98	595.258	5
23	2619.120	n.a.	n.d.	**C**	508.2	-52	508.226	4
24	2706.152	n.a.	n.d.	**S**	348.2	n.a.	348.188	3
25	2820.195	n.a.	n.d.	**N**	261.1	n.a.	261.156	2
26	2948.290	n.a.	n.d.	**K**	n.d.	n.a.	147.113	1

Table S5. ***De novo*** **annotation of the Spat1 trypsin fragment ions, m/z 570.3**

No.	Theor. Mass (m/z)	Mass deviation [ppm]	b-ion (m/z)	AA	y-ion (m/z)	Mass deviation [ppm]	Theor. Mass (m/z)	No.
1	100.0757	n.a.	n.d.	**V**	n.d	n.a.	570.3538	4
2	261.1849	134	261.15	**C**	471.270	-33	471.2854	3
3	424.2483	491	424.04	**Y**	310.200	77	310.1762	2
4	552.3432	24	552.33	**K**	147.100	-87	147.1129	1

Table S6. High-sensitivity amino acid analysis and residue analysis of Spat1

Amino Acid (AA)	nmol/sample	Mole %	AA calc.[#]	% AA calc.
serine	170	15.7	4	16.7
glycine	144	13.4	3	12.5
aspartic acid/asparagine*	95	8.8	2	8.3
glutamic acid/glutamine*	48	4.5	1	4.2
threonine	45	4.2	1	4.2
proline	94	8.7	2	8.3
lysine	91	8.4	2	8.3
tyrosine	88	8.1	2	8.3
valine	128	11.8	3	12.5
isoleucine	131	12.1	3	12.5
phenylalanine	46	4.3	1	4.2
Total	1079	100	24[$]	100

*asparagine is broken down into aspartic acid, and glutamine into glutamic acid; the total amount of these amino acids reported is the combined sum of their individual components; [#]AA, number of amino acids; [$]cysteines and tryptophans are typically not detectable using this technique, hence they were not accounted for in the calculations

Table S7. Similarity of Spat1 amino acid sequence to other UniProt bracelet cyclotides (BLASTp)

Hit	Accession	Description	Organism	Plant family	Length	Identity (%)	E value
**1**	C0HLP2	hyen-H	*Pigea enneasperma*	Violaceae	30	86.2	2.4E-20
**2**	C0HKJ1	mden-I	*Melicytus dentatus*	Violaceae	30	83.3	1.1E-19
**3**	C0HLN8	hyen-D	*Pigea enneasperma*	Violaceae	30	83.3	1.1E-19
**4**	P58433	cycloviolacin-H1	*Viola hederacea*	Violaceae	30	83.3	3.4E-19
**5**	C0HL34	mra3	*Melicytus ramiflorus*	Violaceae	31	82.8	3.8E-19
**6**	C0HLP5	hyen-K	*Pigea enneasperma*	Violaceae	30	80	5E-19
**8**	P86904	cter-Q	*Clitoria ternatea*	Fabaceae	30	80	1.1E-18
**7**	C0HLP0	hyen-F	*Pigea enneasperma*	Violaceae	30	80	1.1E-18
**9**	C0HKK4	vpub-B	*Viola pubescens*	Violaceae	31	82.8	1.2E-18
**10**	C0HKH1	chassatide C6	*Chassalia chartacea*	Rubiaceae	31	82.8	1.2E-18
**11**	C0HKF9	cliotide T6	*Clitoria ternatea*	Fabaceae	30	79.3	1.6E-18
**12**	C0HKI7	mden-E	*Melicytus dentatus*	Violaceae	30	80	2.3E-18
**13**	C0HLN9	hyen-E	*Pigea enneasperma*	Violaceae	30	76.7	2.3E-18
**14**	P58442	cycloviolacin-O10	*Viola odorata*	Violaceae	30	80	3.4E-18
**15**	C0HKJ6	mden-N	*Melicytus dentatus*	Violaceae	31	82.8	5.5E-18
**17**	C0HKJ5	mden-M	*Melicytus dentatus*	Violaceae	31	82.8	8.1E-18
**16**	C0HKK5	vpub-C	*Viola pubescens*	Violaceae	31	79.3	8.1E-18
**19**	C0HKH0	chassatide C9	*Chassalia chartacea*	Rubiaceae	30	80	1.1E-17
**18**	C0HLP1	hyen-G	*Pigea enneasperma*	Violaceae	30	76.7	1.1E-17
**20**	C0HKH2	chassatide C5	*Chassalia chartacea*	Rubiaceae	31	79.3	1.2E-17
**21**	P84639	cycloviolin-C	*Leonia cymosa*	Violaceae	30	80	1.6E-17
**22**	C0HKJ4	mden-L	*Melicytus dentatus*	Violaceae	31	79.3	1.7E-17

**24**	P84641	circulin-C	*Chassalia parviflora*	Rubiaceae	30	80	2.3E-17
**23**	C0HKK0	vdif-A	*Viola diffusa*	Violaceae	30	76.7	2.3E-17
**25**	P86847	cter-G	*Clitoria ternatea*	Fabaceae	30	76.7	3.3E-17
**26**	P85246	vibi-H	*Viola biflora*	Violaceae	31	82.1	3.7E-17
**27**	P58436	cycloviolacin-O4	*Viola odorata*	Violaceae	30	80	4.9E-17
**28**	P86902	cter-P	*Clitoria ternatea*	Fabaceae	30	76.7	4.9E-17
**29**	C0HKJ2	mden-J	*Melicytus dentatus*	Violaceae	31	75.9	5.4E-17
**30**	P83840	vitri peptide A	*Viola arvensis*	Violaceae	30	80	7.1E-17
**31**	C0HKI9	mden-G	*Melicytus dentatus*	Violaceae	30	76.7	7.1E-17
**32**	G1CWH4	cliotide T5 (partial)	*Clitoria ternatea*	Fabaceae	95	80	7.2E-17
**33**	P58435	cycloviolacin-O3	*Viola odorata*	Violaceae	30	80	1E-16
**34**	P85245	vibi-G	*Viola biflora*	Violaceae	31	82.1	1.2E-16
**35**	C0HKJ3	mden-K	*Melicytus dentatus*	Violaceae	31	79.3	1.2E-16
**36**	C0HK40	mech-6	*Melicytus chathamicus*	Violaceae	31	75.9	1.2E-16
**37**	C0HKG0	cliotide T9	*Clitoria ternatea*	Fabaceae	117	80	1.3E-16
**38**	C0HL32	mra1	*Melicytus ramiflorus*	Violaceae	29	80	1.4E-16
**39**	P85244	vibi-F	*Viola biflora*	Violaceae	31	79.3	1.7E-16
**40**	P58456	kalata B5	*Oldenlandia affinis*	Rubiaceae	30	78.6	2.2E-16
**41**	C0HKK3	vpub-A	*Viola pubescens*	Violaceae	31	75.9	2.5E-16
**42**	P86848	cter-H	*Clitoria ternatea*	Fabaceae	30	73.3	3.3E-16
**43**	P84637	cycloviolin-A	*Leonia cymosa*	Violaceae	31	75.9	3.6E-16
**44**	P86846	cter-F	*Clitoria ternatea*	Fabaceae	30	78.6	4.8E-16
**45**	P85180	cycloviolacin-O17	*Viola odorata*	Violaceae	30	76.7	4.8E-16

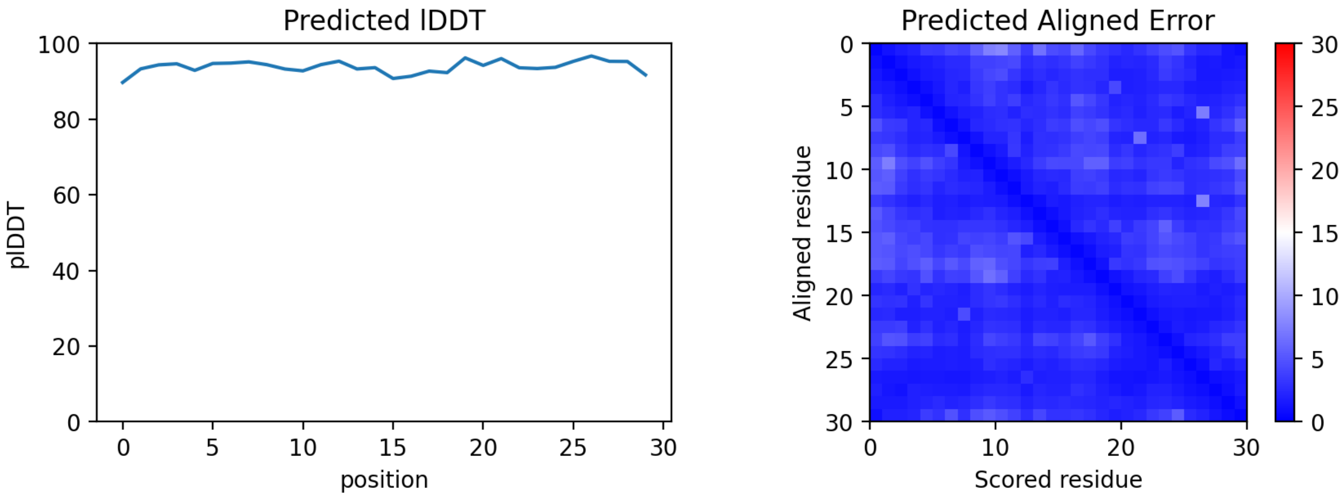

Figure S4. Prediction score of Spat1 using AFCycDesign cyclic peptide modelling. A higher pIDDT value (>90) indicates higher confidence. The Predicted Aligned Error (PAE) heatmap plot displays a very low alignment error (diagonal right blue heatmap), indicating high local structure confidence as well as global peptide arrangement. Overall, high pIDDT and low PAE inform about the reliability of both local structure and domain arrangement.

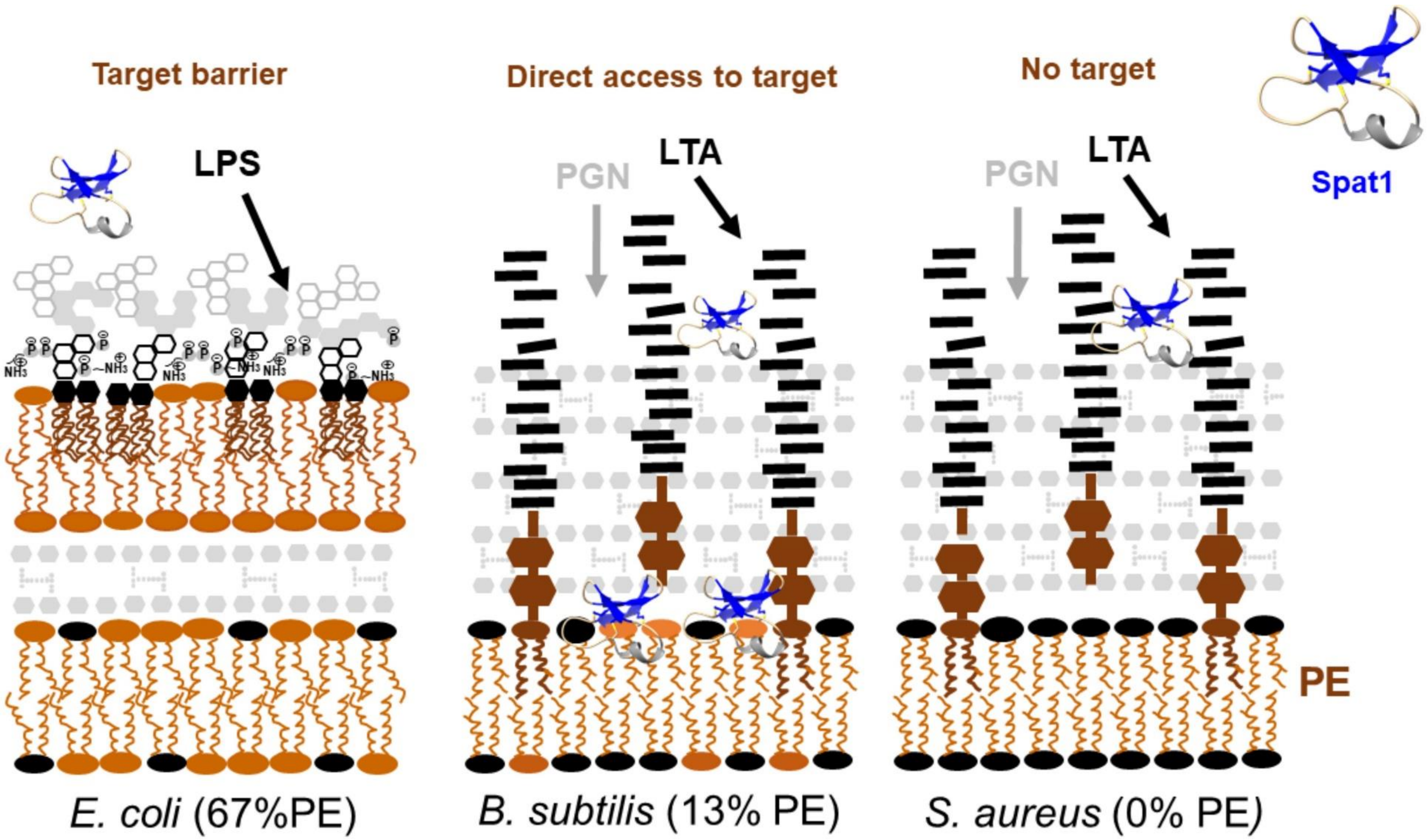

Figure S5. Proposed membrane interaction scheme of Spat1. Spat1's antibacterial activity against Gram-positive bacteria like *B. subtilis* relies on its specific targeting of the accessible phosphatidylethanolamine (PE), unlike the lipopolysaccharide (LPS)-containing outer membrane barrier of Gram-negative *E. coli*. This PE recognition, combined with the peptide's amphipathic structure and hydrophobic patches, facilitates membrane penetration and disruption.
